# Analysis of factors influencing dengue prevention KAP among urban residents in Guangzhou and evaluation of mHealth intervention effects

**DOI:** 10.3389/fpubh.2025.1686267

**Published:** 2025-11-07

**Authors:** Zhihui Huang, Zhaohong Li, Lifen Li, Shiqi Wu, Changming Chen, Nengjiu Li, Jianyun Lu

**Affiliations:** 1 Guangzhou Baiyun District Center for Disease Control and Prevention, Guangzhou, China; 2 Guangzhou Huadu District Huadong Town Health Center, Guangzhou, China

**Keywords:** dengue, mobile health education, intervention, knowledge, attitude, practice (KAP)

## Abstract

**Objective:**

This study aims to analyze the factors influencing dengue fever prevention knowledge, attitude, and practice (KAP) among urban residents in Guangzhou and to evaluate the effectiveness of an mHealth intervention based on the KAP model.

**Methods:**

A cross-sectional study was conducted with 3,571 participants from an online survey. A quasi-experimental study (84 in the intervention group and 83 in the control group) was carried out over 2 months. Electronic questionnaires assessed baseline KAP levels, and the groups’ differences were compared post-intervention.

**Results:**

The baseline survey revealed a knowledge awareness rate of 80.29%, with the lowest levels found in males aged 55 and above, low-education groups, and temporary housing residents. Significant demographic differences were observed in prevention motivation and behavior (*p* < 0.05). Post-intervention, the experimental group showed significant improvements in knowledge scores (*p* < 0.05), but no significant differences were found in prevention motivation (13.26 ± 1.92 vs. 13.32 ± 2.02) and behavior (4.08 ± 1.00 vs. 4.34 ± 0.91) (*p* > 0.05).

**Conclusion:**

The mHealth intervention effectively improved knowledge, but had limited impact on belief formation and behavior change. Future interventions should integrate community-specific strategies to enhance behavioral change mechanisms.

## 1 Introduction

Dengue fever, a mosquito-borne viral illness, is increasingly recognized as a significant global health issue (1). It is estimated by the World Health Organization (WHO) that 390 million infections of dengue are experienced annually, and many of these infections progress to clinical manifestations (2). Dengue exists in over 100 countries, predominantly in the tropical and subtropical regions of the globe (3), where the *Aedes aegypti* mosquito serves as the vector (4). The rapid growth in the incidence of dengue fever is attributable to a synergy of various factors spanning urbanization, climate change, and increased mobility of populations (5). Urban areas characterized by inadequate solid waste management (particularly water-holding containers), suboptimal water storage practices, and elevated human population density demonstrate significantly higher risks of dengue virus transmission (6). This has fueled the increasing frequency of outbreaks, which in turn heightens the public health burden. In cities such as Guangzhou, the frequency of dengue outbreaks has increased, making it essential to develop effective prevention strategies (7).

While dengue vaccines have been available since 2015, vector control remains the principal intervention against dengue fever (8). One of the primary ways of promoting public health and managing dengue dissemination involves ascertaining how the knowledge, attitudes, and practices (KAP) of the inhabitants influence their preventive behavior (9). The most prevalent approach for assessing health behavior is the KAP model, which is founded on the principle that knowledge directly influences attitudes, which in turn influence behavior (10). While the majority of the KAP studies have been applied in chronic illnesses such as diabetes, HIV and coronary heart disease (11–13), fewer studies have applied the KAP model to infectious diseases such as dengue fever. Lifestyle modification represents a pivotal component in the management of cardiovascular disease. The adoption of healthy behaviors is influenced by multiple factors, including knowledge, attitudes, and practices (KAP), social support, and individual patient characteristics. The KAP model provides a valuable framework for conceptualizing and implementing behavioral change. By addressing the underlying psychological determinants of behavior, healthcare providers can more effectively facilitate the adoption of healthy lifestyles in patients (14). Similarly, by evaluating the knowledge of residents about dengue, attitudes toward prevention, and practice, public health campaigns can be more effectively tailored to the population’s needs. Furthermore, the model may be utilized to identify knowledge gaps and misconceptions that may hinder the adoption of preventive behaviors, a necessary step in the creation of effective health interventions.

In recent years, mobile health (mHealth) has emerged as a powerful mechanism for disseminating health information and promoting behavior change (15). mHealth involves the use of mobile technologies such as smartphones and tablets to deliver health-related services, including educational content, reminders, and behavioral interventions (16). The pervasiveness of mobile devices presents a unique opportunity to reach large populations at minimal cost. Research indicates that web-based health interventions demonstrate cost-effectiveness, offering novel perspectives for optimizing public health resource allocation (17). In dengue prevention, mHealth interventions have the potential to spread timely, correct information about the disease, its transmission, and prevention while promoting positive behavior change in the use of insect repellents, elimination of mosquito breeding sites, and avoidance of periods of peak mosquito activity. These interventions can also provide personalized recommendations, making them more effective through customization to individual tastes and needs (18). Previous studies have established that mHealth interventions can successfully impact health knowledge and behaviors in a variety of topics in public health, such as smoking, chronic disease management, and infectious disease prevention (19–22). However, if mHealth is to be effective in dengue prevention, it must not only bridge the knowledge gap but also influence the attitudes and behaviors of the residents in order to prompt them into action.

The aim of this study is to assess the baseline knowledge, attitude, and practice (KAP) levels of dengue fever prevention among the urban residents of Guangzhou. It also seeks to establish the impact of an mHealth intervention, based on the KAP model, in improving the KAP levels, particularly in improving the knowledge and preventive practices of the residents. By targeting specific factors that influence dengue prevention, this study aims to guide the development of more targeted and efficacious health education interventions that can be implemented in Guangzhou, and in other comparable cities with the same public health concerns.

## 2 Methods

### 2.1 Study design and participants

This study employed a two-phase, sequential design, comprising an initial cross-sectional survey followed by a quasi-experimental intervention.

#### 2.1.1 Phase 1: cross-sectional survey

A cross-sectional survey was conducted to assess the baseline levels of knowledge, attitudes, and practices (KAP) regarding dengue prevention. Participants were recruited using a convenience sampling method. Specifically, the study distributed an online questionnaire through WeChat communities (e.g., resident groups, workplace groups) targeting permanent residents of Guangzhou. The survey was administered between September 20, 2024, and October 20, 2024, and the final cross-sectional sample consisted of 3,571 permanent residents of Guangzhou aged 18 to 70 years.

#### 2.1.2 Phase 2: quasi-experimental study

Following the survey, a subset of participants was invited to join an mHealth intervention study. The eligibility criteria for this phase included: (1) being a permanent resident of Guangzhou; (2) aged 18–70 years; (3) ability to use WeChat; (4) having completed the baseline survey; (5) agreed to join the WeChat group and be assigned to a group. A total of 167 individuals voluntarily agreed to participate and were allocated into groups based on self-selection. Participants self-selected into either the intervention group or the control group.

### 2.2 Questionnaire development

The data collection instrument was a self-administered questionnaire based on the knowledge, attitude, and practices (KAP) model. Initial items were generated from a comprehensive review of relevant literature on dengue prevention. Content validity was established through consultations with a panel of five experts in public health and infectious diseases. The final questionnaire comprised 29 items across three core domains: knowledge (e.g., transmission routes, symptoms), attitudes (e.g., perceived severity, susceptibility), and practices (e.g., mosquito breeding site elimination, personal protective behaviors). Demographic information was also collected.

### 2.3 Questionnaire testing and reliability

Prior to the formal survey, the questionnaire was distributed to 90 residents through WeChat communities in a pilot test to assess clarity, comprehensibility, and feasibility. All questionnaires were returned (100% response rate). The internal consistency reliability of the questionnaire was evaluated using Cronbach’s alpha coefficient based on the pilot data. The overall Cronbach’s *α* for the questionnaire was 0.806, indicating good reliability.

### 2.4 Intervention details

The intervention group received a comprehensive mHealth intervention over 2 months. This included:

#### 2.4.1 Personalized knowledge delivery

Students were given dengue-specific learning content, including symptoms, transmission modes, and prevention methods, according to their starting knowledge level.

#### 2.4.2 Interactive engagement

The intervention comprised quizzes, polls, and WeChat discussions in order to enhance learning and provide continuous feedback.

#### 2.4.3 Behavioral guidance

The participants were encouraged to adopt some preventive practices such as draining stagnant water, sleeping in insect-repellent coated mosquito nets, and using insect repellent.

#### 2.4.4 Community support

Intervention enabled peer education and community engagement through the creation of a WeChat group where members educated each other and shared mutual support to take dengue prevention measures.

The control group received basic health education, including information on dengue symptoms and treatment options, but did not participate in the interactive and behavioral components of the intervention.

### 2.5 Data collection

Data were collected by electronic questionnaires at two time points: baseline (before intervention) and after the two-month intervention. The questionnaires assessed knowledge, attitudes, and practices for prevention of dengue. Multiple-choice questions were used to assess knowledge, a Likert scale to assess attitudes, and self-reported preventive acts to assess practices.

### 2.6 Statistical analysis

Data were analyzed using SPSS 24.0 software. Descriptive statistics were used to report demographic variables and KAP scores. Baseline characteristics across different groups in the cross-sectional survey were compared using *t*-tests, ANOVA, Chi-square, or Fisher’s exact tests as appropriate. The effect of the intervention was assessed by comparing pre- and post-intervention scores within and between the two groups with paired t-tests for continuous variables and chi-square tests for categorical variables. *p* < 0.05 was used as the level of statistical significance.

### 2.7 Ethics approval

This study was approved by the Ethics Committee of the Guangzhou Baiyun District Center for Disease Control and Prevention. All collected data were anonymized, and no personally identifiable information was obtained.

#### 2.7.1 Baseline survey

Before accessing the online survey, all potential participants were presented with an electronic informed consent form. This form outlined the study’s objectives, procedures, voluntary participation and the confidentiality measures. Participants under 18 years of age were required to obtain parental or guardian approval before agreeing.

#### 2.7.2 mHealth intervention trial

Eligible adults were recruited exclusively from the baseline cohort. Participation in the mHealth intervention phase commenced upon voluntary enrollment into designated WeChat groups. Participants retained the right to withdraw from the intervention at any time by exiting the WeChat group.

## 3 Results

### 3.1 Baseline survey

#### 3.1.1 Among the knowledge awareness

The overall dengue knowledge awareness rate was 80.29%. Significant differences were found across age, gender, residential area, housing type, education level, and occupation (*p* < 0.05). For instance, residents aged 35–54 showed the highest awareness (82.49%), while those aged 55 and above had the lowest (64.44%). The results as shown in Table 1.

**Table 1:** Comparison of dengue knowledge awareness rate among different demographic groups.

Categorical variable	Group	Number of participants (*n* = 3,571)	Awareness rate (%)	*χ* ^2^	*p*-value
Age	18–34 years	1,368	80.48	51.001	<0.05
	35–54 years	1919	82.49		
	≥55 years	284	64.44		
Gender	Female	2,475	81.54	7.957	<0.05
	Male	1,096	77.46		
Residential area	High-density population	2,434	83.98	65.811	<0.05
	Low-density population	1,137	72.38		
Housing type	Self-built (rural/suburban)	1775	80.85	38.831	<0.05
	Urban village residence	471	84.71		
	Multi-story walk-up	56	75.00		
	High-rise elevator	518	80.31		
	High-rise elevator	669	78.03		
	Temporary housing	17	29.41		
	Others	65	73.85		
Education level	Primary school or below	49	53.06	187.881	<0.05
	Junior high school	498	62.05		
	High school/vocational	762	76.12		
	Bachelor’s/Associate	2,221	86.36		
	Master’s or above	41	82.93		
Occupation	Student	44	79.55	65.892	<0.05
	Teacher	431	83.53		
	Catering staff	94	74.47		
	Public service attendant	306	83.66		
	Business professional	285	84.56		
	Worker/Migrant laborer	736	80.03		
	Medical staff	137	97.81		
	Civil servant	299	83.28		
	Retiree	172	68.02		
	Homemaker/Unemployed	778	77.25		
	Other occupations	289	74.39		
History of dengue	Yes	41	80.49	0.001	0.974
	No	3,530	80.28		
Total		3,571	80.29		

#### 3.1.2 Among the prevention motivation

Most residents held positive attitudes toward dengue severity, susceptibility, and prevention. However, significant differences were observed across education levels and occupations (*p* < 0.05). The results as shown in Table 2.

**Table 2:** Comparison of dengue prevention motivation among different population groups.

Categorical variable	Group	Mean	SD	Statistic (*F*/*t*)	*p*-value
Age	18–34 years	6.99	2.05	0.569^a^	0.566
	35–54 years	7.07	1.94		
	≥55 years	7.07	2.07		
Gender	Female	7.00	1.93	-1.491^b^	0.136
	Male	7.12	2.12		
Residential area	High-density population area	7.08	2.04	1.741^b^	0.082
	Low-density population area	6.95	1.89		
Housing type	Self-built (rural/suburban)	7.02	1.99	1.836^a^	0.095
	Urban village residence	7.09	1.95		
	Low-rise self-built (urban)	7.61	1.79		
	Multi-story walk-up	7.15	2.10		
	High-rise elevator	6.90	1.98		
	Temporary housing	7.24	1.89		
	Others	7.17	1.72		
Education level	Primary school or below	7.55	2.49	6.726^a^	<0.05
	Junior high school	7.34	1.87		
	High school/vocational	7.18	2.02		
	Bachelor’s/Associate	6.92	1.97		
	Master’s or above	6.88	2.71		
Occupation	Student	7.66	2.28	3.682^a^	<0.05
	Teacher	6.93	1.75		
	Catering staff	6.94	2.02		
	Public service attendant	7.12	2.00		
	Business professional	6.80	1.96		
	Worker/Migrant laborer	7.12	2.01		
	Medical staff	6.36	1.87		
	Civil servant	6.92	1.95		
	Retiree	6.90	2.06		
	Homemaker/Unemployed	7.21	2.06		
	Other occupations	7.15	2.05		
History of dengue	Yes	6.61	2.04	-1.385^b^	0.166
	No	7.04	1.99		

^a^One-way ANOVA for ≥3 groups; ^b^Independent samples *t*-test for two groups.

#### 3.1.3 Among the prevention behavior

The average prevention behavior score was 15.05 ± 3.38. Significant differences were found across various demographic groups (*p* < 0.05). The results as shown in Table 3.

**Table 3:** Comparison of dengue prevention behavior scores among different population groups.

Categorical variable	Group	Mean	SD	Statistic (*F*/*t*)	*p*-value
Age	18–34 years	15.04	3.38	29.103^a^	<0.05
	35–54 years	15.29	3.25		
	≥55 years	13.49	3.79		
Gender	Female	15.19	3.26	3.558^b^	<0.05
	Male	14.74	3.61		
Residential area	High-density population area	15.29	3.32	6.121^b^	<0.05
	Low-density population area	14.54	3.45		
Housing type	Self-built (rural/suburban)	15.27	3.29	6.455^a^	<0.05
	Urban village residence	15.32	3.14		
	Low-rise self-built (urban)	14.09	3.85		
	Multi-story walk-up	14.88	3.43		
	High-rise elevator	14.66	3.47		
	Temporary housing	11.12	4.26		
	Others	14.51	4.17		
Education level	Primary school or below	13.18	4.58	29.133^a^	<0.05
	Junior high school	13.72	3.71		
	High school/vocational	14.81	3.58		
	Bachelor’s/Associate	15.49	3.08		
	Master’s or above	14.61	3.60		
Occupation	Student	14.73	3.47	7.357^a^	<0.05
	Teacher	15.76	2.94		
	Catering staff	14.67	3.39		
	Public service attendant	15.68	3.21		
	Business professional	14.98	3.41		
	Worker/Migrant laborer	14.98	3.50		
	Medical staff	16.00	3.01		
	Civil servant	15.15	3.35		
	Retiree	14.23	3.62		
	Homemaker/Unemployed	14.80	3.28		
	Other occupations	14.40	3.73		
History of dengue	Yes	16.44	2.75	2.643^b^	<0.05
	No	15.04	3.38		
Total		15.05	3.38		

^a^One-way ANOVA for ≥3 groups; ^b^Independent samples *t*-test for two groups.

### 3.2 Intervention effectiveness

A total of 167 questionnaires were distributed online for the quasi-experimental phase. After excluding invalid responses, we obtained 150 valid questionnaires, achieving a response rate of 89.82%. The intervention group comprised 74 participants, while the control group included 76 participants. The two groups were largely comparable at baseline, with no statistically significant differences were observed in most demographic characteristics. The results as shown in Table 4.

**Table 4:** Comparison of baseline characteristics between the intervention and control groups.

Characteristic	Category	Intervention group (*n* = 74)	Control group (*n* = 76)	Statistical value	*p*-value
Age (years)	Mean ± SD	35.64 ± 8.76	34.22 ± 6.08	*t* = 1.143	0.255
Gender	Female	61(82.4%)	63(82.9%)	*χ*^2^ = 0.006	0.940
	Male	13(17.6%)	13(17.1%)		
Residential area	High-density population	16(21.6%)	12(15.8%)	*χ*^2^ = 0.840	0.359
	Low-density population	58(78.4%)	64(84.2%)		
Housing type	Self-built (rural/suburban)	33(44.6%)	46(60.5%)	FET	0.191
	Urban village residence	10(13.5%)	6(7.9%)		
	Multi-story walk-up	13(17.6%)	8(10.5%)		
	High-rise elevator	17(23.0%)	12(15.8%)		
	Temporary housing	0(0.0%)	1(1.3%)		
	Others	1(1.4%)	3(3.9%)		
Education level	Junior high school	12(16.2%)	18(23.7%)	U = 2535.000	0.247
	High school/vocational	18(24.3%)	19(25.0%)		
	Bachelor’s/Associate	44(59.5%)	39(51.3%)		
Occupation	Teacher	4(5.4%)	5(6.6%)	FET	*p* < 0.05
	Catering staff	3(4.1%)	1(1.3%)		
	Public service attendant	5(6.8%)	5(6.6%)		
	Business professional	4(5.4%)	11(14.5%)		
	Worker/Migrant laborer	10(13.5%)	8(10.5%)		
	Medical staff	16(21.6%)	3(3.9%)		
	Civil servant	4(5.4%)	6(7.9%)		
	Retiree	4(5.4%)	1(1.3%)		
	Homemaker/Unemployed	19(25.7%)	27(35.5%)		
	Other occupations	5(6.8%)	9(11.8%)		

Data are presented as *n* (%) or mean ± standard deviation. FET, Fisher’s exact test.

The intervention group showed a significant increase in knowledge scores post-intervention (*p* < 0.05), with scores rising from 5.84 ± 1.21 to 6.46 ± 0.95. The results as shown in Table 5.

**Table 5:** Comparison of knowledge dimension scores before and after intervention.

Group	Before intervention	After intervention	Statistic	*p*-value
Intervention	5.84 ± 1.21	6.46 ± 0.95	*t* = −3.479	<0.05
Control	5.20 ± 1.40	4.99 ± 1.05	*t* = 1.05	0.295

No significant differences were observed in prevention motivation scores between the two groups post-intervention (*p* > 0.05). No significant differences were observed in prevention behavior scores between the two groups post-intervention (*p* > 0.05). The results as shown in Tables 6, 7.

**Table 6:** Comparison of belief dimension scores before and after intervention.

Group	Before intervention	After intervention	Statistic	*p*-value
Intervention	13.26 ± 1.92	13.32 ± 2.02	*t* = −0.209	0.835
Control	13.16 ± 1.84	12.99 ± 1.84	*t* = 0.572	0.568

**Table 7:** Comparison of behavior dimension scores before and after intervention.

Group	Before intervention	After intervention	Statistic	*p*-value
Intervention	4.08 ± 1.00	4.34 ± 0.91	*t* = −1.63	0.105
Control	3.86 ± 0.93	4.17 ± 1.01	*t* = −1.999	0.047

## 4 Discussion

### 4.1 The factors influencing dengue fever prevention KAP

#### 4.1.1 Knowledge levels and demographic variations

The baseline survey discovered that the overall knowledge level of dengue prevention among Guangzhou residents was relatively high (80.29%). Substantial variations exist in the awareness and knowledge of dengue fever among diverse geographical regions, with Europe exhibiting the highest levels of knowledge and Africa exhibiting the lowest (9). This suggests that the awareness level of dengue fever prevention and control knowledge among Guangzhou residents is relatively high, possibly because dengue outbreaks occur frequently in Guangdong Province, prompting the local government to place a greater emphasis on health education. This survey was conducted during a dengue outbreak in this area. In response to such outbreaks, local entities initiated emergency health education programs, thereby increasing the frequency of household health education for individuals residing in impacted regions. As a result, the respondents were able to acquire relevant knowledge relatively well within a certain period. There were, however, significant demographic disparities. Older residents (aged 55 and above), males, low-density population areas, retirees and less educated individuals had lower knowledge scores.

Similarly, the age group of 35–54 years exhibited the highest levels of awareness. This observation aligns with the findings of Kumari (23), who reported consistent age-related patterns in the Indian population. In contrast, adults aged 18–34 years demonstrated lower awareness of dengue, likely due to their familial and occupational responsibilities (24). Furthermore, their knowledge appears to be constrained by their level of educational attainment (25). Notably, individuals aged ≥55 years exhibited the lowest awareness rates, primarily due to barriers in accessing health information, including gaps in digital literacy.

Women exhibited higher levels of awareness than men. This observation is consistent with the conclusion that women generally possess superior knowledge and practices regarding mosquito control. Some studies have proposed that social roles, such as women’s increased responsibility for household sanitation, may contribute to this disparity in knowledge (26). Moreover, women represent a significant demographic on social media platforms such as WeChat, and their heightened interest in accessing health information relative to men may lead to increased engagement rates (27).

Epidemiological information, health literacy, and policy interventions are more easily disseminated in high-density areas, as dense surveillance systems and public facilities accelerate the diffusion of information (28). Television, radio, and social media were the main sources of information in the dengue study; media coverage was more concentrated in high-density areas (29, 30). In addition, high-density zones have better healthcare systems and community services, which allows for more efficient implementation of health promotion and social interventions, indirectly contributing to the reach of information (31). Urban villages in China represent high-risk zones for dengue transmission owing to inadequate governance and high population mobility. This highlights the critical need for targeted public health interventions in densely populated areas. Dengue fever disease in the main urban area of Guangzhou mainly manifests itself in the gradual spread of dengue fever disease from urban villages and old urban areas with poorer sanitary environment to the surrounding new urban areas (32). Due to government attention, residents in these high-risk areas are more likely to receive pertinent health information and preventive measures during periods of heightened dengue risk, thereby increasing awareness.

Higher education levels are associated with higher levels of dengue awareness, and literacy may be a key determinant of dengue awareness. This study shows that the level of health awareness is related to literacy; the higher the literacy level of the residents, the more attention they pay to health-related information, the more initiative they take in searching for health information, and the more diverse their sources of information. The lower the literacy level of residents, the more restricted the cognitive pathways of health education, resulting in a lower level of health awareness. Educational level independently influences health cognitive abilities, with highly literate residents being better at understanding and utilizing health information (33). The knowledge level of dengue fever also varied among occupational groups. The knowledge rate of medical personnel was significantly higher than that of other occupational groups (34), which is in line with the Ethiopian study and suggests that the resources of medical personnel should be fully utilized to disseminate dengue-related knowledge. In this study, we found that retired people had the lowest awareness of dengue fever, which may be related to their reliance on traditional media (TV/newspapers) but with low frequency of exposure and inadequate use of digital tools (35). To increase awareness and reduce the risk of infection, a combination of measures, such as strengthening health education campaigns, broadening access to information, and increasing social participation, are needed.

The demographic determinants of knowledge indicate the necessity for particularly targeting subgroups in health education campaigns. For example, older individuals may require special assistance and information to increase their knowledge and openness to prevention initiatives. Similarly, those with lower education levels may enjoy simpler messages emphasizing the short-term benefits of prevention efforts against dengue. Recognizing women’s high engagement in social media health dissemination presents significant public health opportunities. Leverage this to enhance health literacy and outcomes for women and families, maximizing social media’s public health value.

#### 4.1.2 Motivation for dengue fever prevention

In this study, there were differences in the motivation to prevent dengue fever between people with different literacy levels and occupations, mainly in the form of higher scores of motivation to prevent in elementary school and below, and in occupations as students. Lower educated people pay more attention to prevention due to intuition, while higher educated people may underestimate the risk due to cognitive bias (36). Therefore, more in-depth and professional health education campaigns should be carried out for the more educated population, emphasizing the dangers of dengue fever and the importance of preventive measures, and improving their knowledge of dengue fever and their practical ability through scientific data and cases.

Motivation for dengue prevention also varied among different occupational groups. The motivation for prevention was significantly higher among students than among other occupational groups, which may be due to the fact that schools, as an important place for knowledge dissemination, develop students’ health awareness through classroom teaching and practical activities, leading to enhanced dengue prevention consciousness.

#### 4.1.3 Dengue fever prevention behaviors

In this study, variations in dengue prevention behavior scores were observed among individuals of different ages, genders, residential areas, housing types, literacy levels, occupational categories, and prior experiences with dengue fever. Notably, lower levels of preventive behaviors were identified among residents aged 55 years and older, males, those residing in low-density populated areas, individuals living in temporary housing facilities, those with an education level of elementary school or below, retirees, and those who had not previously contracted dengue fever (37, 38). Compared to other age groups, individuals aged 55 years and above exhibited lower levels of dengue prevention behaviors, potentially attributable to factors such as historical environmental influences, a relative lack of health awareness within this demographic, and inadequate hygiene practices in their daily lives, which collectively contributed to their reduced mosquito prevention behaviors. Furthermore, the preventive behavior scores of females were higher than those of males, aligning with the findings of Ahmed (39).

### 4.2 Impact of mHealth intervention

Mobile text messaging is an effective, acceptable, and appropriate health intervention that can improve community dengue prevention practices (40). Although the SMS function is similar to WeChat’s information push, our research emphasizes community interaction, which is also a key mechanism for knowledge improvement. This study explored the impact of information, education, and communication interventions on dengue knowledge, supporting the effectiveness of mobile health interventions, with community interaction being central to knowledge improvement (41).

This study, based on the KAP model, demonstrated that WeChat mobile health interventions, through 2 months of targeted information delivery and community interaction, significantly improved community residents’ knowledge of dengue fever (*p* < 0.05). The mHealth intervention significantly improved knowledge in the intervention group, being evidence of effectiveness of mobile platforms in health information. The findings validate previous research that has proven mHealth interventions to successfully improve knowledge among different populations (42). This result is consistent with the effectiveness of mobile health education in the management of chronic diseases (15, 43). This study further confirms that combining multiple formats, such as popular science articles and graphics, video explanations, and expert consultations, can effectively overcome the communication bottlenecks of traditional health education (44, 45).

Behavior change theories suggest that interventions addressing both knowledge and motivational factors are needed to produce lasting behavior change. The KAP model operates on the assumption that increased knowledge directly leads to changes in attitudes and subsequent behavior (46, 47). In reality, behavioral change may be significantly moderated by external factors such as the social environment, cultural context, and economic conditions. Furthermore, the KAP model primarily focuses on individual-level determinants while overlooking the influence of macro-level factors, including policy, institutional frameworks, and the broader environment.

A study conducted in Cambodia revealed that despite participants demonstrating relatively high knowledge levels regarding dengue fever, their actual preventive behaviors did not show significant improvement (48). However, in this study, the effects of the intervention on belief restructuring and behavioral transformation were limited, which may have been influenced by factors such as the intervention strategies, characteristics of the target population, and the social environment. In a study conducted in Indonesia, although knowledge about the dengue vaccine among community members and healthcare providers improved, changes in beliefs and behaviors remained limited (49). Similarly, research in Malaysia indicates that despite a high level of knowledge, residents’ preventive behaviors have not significantly improved, suggesting that increased awareness does not directly translate into changes in behavior (50). This outcome highlights the complexity involved in effectively translating knowledge into practical application.

Further analysis shows that the interaction between sociocultural contexts and individual psychological mechanisms increases the difficulty of transforming knowledge into action. Traditional cognitive frameworks significantly influence the processing of new information at the level of beliefs. For instance, although some residents recognize that eliminating stagnant water can disrupt mosquito breeding, they are swayed by the stereotype that “dengue fever is a disease of tropical slums,” leading them to believe that their own living environment does not necessitate preventive measures. This cognitive bias closely resembles the phenomenon observed in Cambodia, where there is a misalignment between residents’ knowledge levels and their prevention practices (48).

In summary, although mobile health interventions based on the KAP model are effective in increasing knowledge levels, additional strategies are still needed to achieve comprehensive behavior change in terms of beliefs and actions.

### 4.3 Limitations and future directions

There are some limitations of this research. First, the non-randomized, self-selection group allocation may introduce selection bias. Second, the sample of internet and mobile phone users may limit generalizability to populations with lower digital literacy (51). Third, the two-month follow-up period prevents assessment of long-term behavior change. Finally, the use of basic statistical tests without controlling for potential confounding factors may affect the precision of the effect estimates.

Subsequent research should employ randomized controlled designs to mitigate selection bias, broaden sampling to include underrepresented groups, utilize advanced statistical models(e.g., ANCOVA) to control for confounders, and incorporate long-term follow-up to evaluate the sustainability of behavior changes. Additionally, collaboration with community-based organizations and local health authorities can enhance the effectiveness of mHealth interventions in real-world settings.

## 5 Conclusion

This study showed that an mHealth intervention grounded in the KAP model was successful in improving dengue prevention knowledge among Guangzhou’s urban dwellers. The intervention, though, failed to make a significant change in attitudes and behaviors, illustrating the intricacies of behavior change in public health.

To realize maximum impact of mHealth interventions, future strategies must address not just knowledge gaps but also emotional and behavioral determinants of health behavior. With personalized content, interactive learning, and community engagement, mHealth interventions can play a major role in reducing the burden of dengue and other vector-borne diseases in urban settings.

Continued study is needed to create these interventions and identify ways to facilitate long-term behavior changes, with research translating into ongoing preventive actions.

### 5.1 Innovation

This study provides baseline data for dengue prevention and control by identifying knowledge gaps and low-compliance populations. It also explores the integration of mHealth intervention with KAP theory, offering new insights for infectious disease prevention.

## Data Availability

The raw data supporting the conclusions of this article will be made available by the authors, without undue reservation.
